# P-1654. Determinants of Severe COVID-19 in Hospitalized Children: Insights from a Multicenter Retrospective Study in Lima, Peru

**DOI:** 10.1093/ofid/ofaf695.1829

**Published:** 2026-01-11

**Authors:** Rafaella Navarro Hoyos, Valerie Mayorga Huallpa, Brian Peña Calero, Theresa Ochoa Woodell

**Affiliations:** Instituto de Medicina Tropical Alexander von Humboldt, Universidad Peruana Cayetano Heredia, Lima, Lima, Peru, Birmingham, Alabama; Instituto de Medicina Tropical Alexander von Humboldt, Universidad Peruana Cayetano Heredia, Lima, Lima, Peru, Birmingham, Alabama; Instituto de Medicina Tropical Alexander von Humboldt, Universidad Peruana Cayetano Heredia, Lima, Lima, Peru, Birmingham, Alabama; Instituto de Medicina Tropical Alexander von Humboldt, Universidad Peruana Cayetano Heredia, Lima, Lima, Peru, Birmingham, Alabama

## Abstract

**Background:**

Peru has experienced the highest number of COVID-19 deaths per million inhabitants worldwide. Although children were less frequently affected than adults, a substantial number have required hospitalization and experienced severe outcomes. This study aimed to identify factors associated with severe COVID-19 among hospitalized children and adolescents in major hospitals in Lima, Peru.

**Figure:**
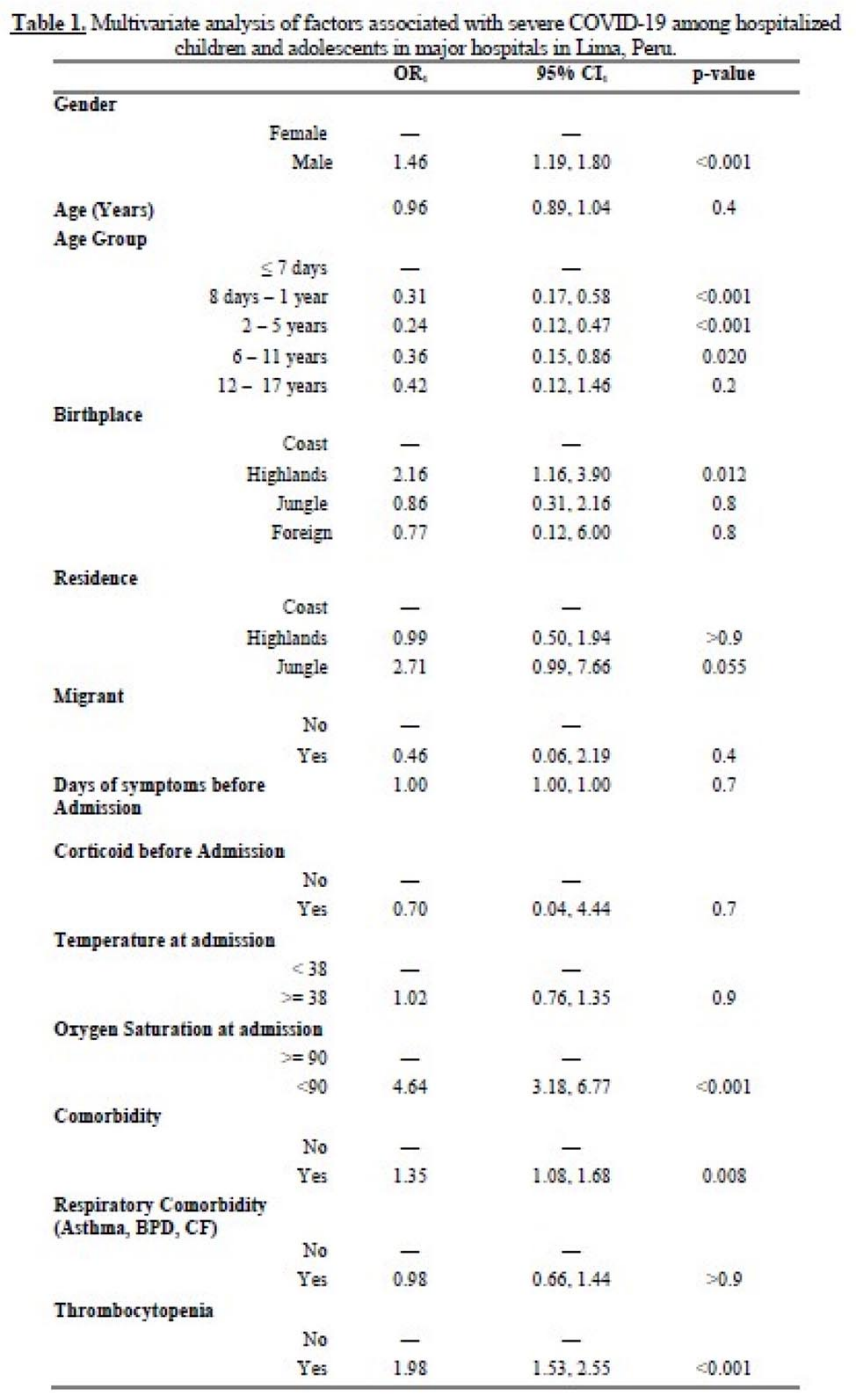
Median (Q1, Q3), 1OR = Odds Ratio, CI = Confidence Interval, BDP = Bronchopulmonary Dysplasia, CF = Cystic fibrosis

**Methods:**

A multicenter retrospective study of children and adolescents under 18 years of age hospitalized due to COVID-19 or multisystem inflammatory syndrome in children (MISC) across 15 hospitals in Lima between March 2020 and February 2023. Severe COVID-19 was defined as the requirement for invasive mechanical ventilation, vasopressor or inotropic support, ECMO, or death. To identify factors associated with severe COVID-19 multivariate logistic regression analysis was performed. Odds ratios (OR) with 95% confidence intervals (CI) were calculated.

**Results:**

A total of 4,179 medical records were included. Males accounted for 51.4% (n = 2,116) and children < 5 years accounted for 50.8% (n = 2,125). Comorbidities were reported in 47.0% (n = 1,962) of cases, with asthma (n = 270), prematurity (n = 211), epilepsy (n = 148) and obesity (n = 123) being the most frequent. The overall mortality rate was 2.6% (n = 106); 641 patients met the severity criteria (15.3%) and 3,538 were non-severe. The main diagnosis were pneumonia 22.7% (46.7% vs 18.4% among severe and non-severe cases), respiratory failure 12.1% (51.1% vs 5.0%), sepsis 9.5% (35.8% vs 4.7%) and MISC 8.9% (19.2% vs 7.0%). In the multivariate analysis, oxygen saturation < 90% at admission (OR = 4.64; 95% CI: 3.18–6.77), being born in the Highlands region (OR = 2.16; 95% CI: 1.16–3.90), thrombocytopenia (OR = 1.98; 95% CI: 1.53–2.55), male sex (OR = 1.46; 95% CI: 1.19–1.80), and presence of comorbidities (OR = 1.35; 95% CI: 1.08–1.68) were significantly associated with severe disease.

**Conclusion:**

Severe COVID-19 in children and adolescents hospitalized in Lima was associated with hypoxemia at admission, being born in the Highlands region, thrombocytopenia, male sex and comorbidities.

**Disclosures:**

Rafaella Navarro Hoyos, MD, Pfizer: Grant/Research Support Valerie Mayorga Huallpa, MD, Pfizer: Grant/Research Support Brian Peña Calero, n/a, Pfizer: Grant/Research Support Theresa Ochoa Woodell, PhD, Pfizer: Grant/Research Support

